# Human Normal Bronchial Epithelial Cells: A Novel *In Vitro* Cell Model for Toxicity Evaluation

**DOI:** 10.1371/journal.pone.0123520

**Published:** 2015-04-10

**Authors:** Wenqiang Feng, Juanjuan Guo, Haiyan Huang, Bo Xia, Hongya Liu, Jie Li, Shaolin Lin, Tiyuan Li, Jianjun Liu, Hui Li

**Affiliations:** 1 State Key Laboratory of Virology, Institute of Medical Virology, Wuhan University School of Basic Medical Sciences, Wuhan, Hubei, China; 2 Shenzhen R&D center of State Key Laboratory of Virology, Wuhan University Shenzhen Institute, Shenzhen, Guangdong, China; 3 Key Laboratory of Modern Toxicology of Shenzhen, Shenzhen Center for Disease Control and Prevention, Shenzhen, Guangdong, China; 4 Shenzhen People's Hospital, Shenzhen, Guangdong, China; Georgetown University, UNITED STATES

## Abstract

Human normal cell-based systems are needed for drug discovery and toxicity evaluation. hTERT or viral genes transduced human cells are currently widely used for these studies, while these cells exhibited abnormal differentiation potential or response to biological and chemical signals. In this study, we established human normal bronchial epithelial cells (HNBEC) using a defined primary epithelial cell culture medium without transduction of exogenous genes. This system may involve decreased IL-1 signaling and enhanced Wnt signaling in cells. Our data demonstrated that HNBEC exhibited a normal diploid karyotype. They formed well-defined spheres in matrigel 3D culture while cancer cells (HeLa) formed disorganized aggregates. HNBEC cells possessed a normal cellular response to DNA damage and did not induce tumor formation in vivo by xenograft assays. Importantly, we assessed the potential of these cells in toxicity evaluation of the common occupational toxicants that may affect human respiratory system. Our results demonstrated that HNBEC cells are more sensitive to exposure of 10~20 nm-sized SiO_2_, Cr(VI) and B(a)P compared to 16HBE cells (a SV40-immortalized human bronchial epithelial cells). This study provides a novel in vitro human cells-based model for toxicity evaluation, may also be facilitating studies in basic cell biology, cancer biology and drug discovery.

## Introduction

The organ-specific differentiated epithelial cells are directly associated with the specific functions of vital organs, such as lung, kidney, liver and skin. These epithelial cells are usually the primary targets of environmental toxicants for damage. Human normal epithelial cell models are required for in vitro studies of interactions between human tissues and environmental toxicants. Usually ones use spontaneously immortalized cell lines (e.g., HaCaT) or viral genes/cellular genes immortalized cell lines for these studies[1–4].

Nanometer silicon dioxide (nano-SiO_2_) has recently become one of the most popular nano-materials in many fields, such as industrial manufacturing, engineering and biomedicine. However, nano-SiO_2_ is easily evaporated into air. It has been shown that inhalation of nano-SiO_2_ causes pulmonary and cardiovascular alteration and damages in old rats [5] and lung fibrogenesis in rats [6]. Hexavalent chromium (Cr (VI)) is widely used in the manufacture of various industrial products, such as stainless steel. Occupational exposure to Cr (VI) may cause respiratory cancer. Studies on chromate production workers indicate that exposure to Cr (VI) was responsible for an increased relative risk of developing lung cancer [7]. Benzo[a]pyrene (B[a]P) is the common toxicant which is present in car exhaust, incomplete fossil fuel combustion, tobacco smoke and occupational exposures. International Agency for Research of Cancer sets B[a]P as Class 2 group A human carcinogen. B(a)P is a known carcinogen to lung, stomach and skin [8, 9].

All of these three toxicants cause damages or even cancer in lung. Studies have been done to investigate the underlying mechanisms. Skin and lung tissue are the potential primary routes and targets of occupational nano-SiO_2_ exposure. It has been shown that nano-SiO_2_ alters the expression of proteins associated with oxidative stress and apoptosis [1] and epigenetic regulation of a cellular repair gene poly(ADP-ribose) polymerases-1(PARP-1) in HaCaT cells [10]. However, HaCaT cells are spontaneously immortalized keratinocytes with an aneuploid chromosome [11] which has the biological properties far from normal. Other studies used cancer cell lines or viral/oncogene immortalized cell lines to investigate the cytotoxicity/genocytoxicity exposed to nano-SiO_2_, Cr(VI) and B(a)P [2–4, 12]. Recent studies have demonstrated that exogenous gene-immortalized cells have an altered genetic background/signaling pathways and exhibit an abnormal biological characters [13, 14]. That is the major reason why studies turn out to be controversial results using cancer cells or gene-immortalized cells as models. Recently, murine fibroblastic 3T3 feeder cells derived from mouse embryo and fetal bovine serum (FBS) are used to promote epithelial cell proliferation in vitro [13, 15]. Ideally, these xenogeneic materials from the culture should be eliminated for human cell based studies, especially for the long term regenerative medicine studies.

In this study, we established human normal bronchial epithelial cells (HNBEC) from the discarded adjacent normal tissue of lung cancer after surgery from a Chinese patient in Shenzhen China. The short tandem repeats (STR) analysis showed that HNBEC cells have 15 loci and the Y-specific Amelogenin locus that do not match any other cell lines published or registered before. We also verified its normal biological features by karyotype analysis, matrigel 3D culture, DNA damage response and xenograft assays. Moreover, we evaluated its potential applications to toxicological model by common occupational toxicants exposure to lung tissue. Our results demonstrated that HNBEC cells were more sensitive to exposure of 10~20nm-sized SiO_2,_ Cr(VI) and B(a)P compare to 16HBE cells (a SV40-immortalized human bronchial epithelial cells). Since HNBEC cells were established without any genetic background changes, the cellular responses to toxicants are real physiological status. HNBEC cells provide a valuable in vitro human cells-based model for toxicological research.

## Materials and Methods

### 1. Cell isolation and propagation

Adjacent normal lung tissue from a surgical specimen of lung tumor was obtained with the written informed consent of the patient. Institutional review boards at Wuhan University Shenzhen Institute and Shenzhen People’s Hospital approved this study. To avoid contamination of tumor tissue for the specimen, the normal tissue should be obtained as far from the tumor lesion as possible. Besides, mirror structure of specimen were separated and sent for histology. Uncontaminated sample was delivered to research lab and tissue was minced and dispersed into single cells by digestion with collagenase (StemCell Technologies Inc, Vancouver, BC, Canada) plus trypsin. Epithelial Cells were then cultured using primary epithelial culture medium (ImmorTech, China), or serum free medium (Life Technologies), or DMEM (Life Technologies) supplemented with 10% FBS (Invitrogen). The primary epithelial culture medium contains DMEM and nutrient F-12 Ham (3:1) (Sigma-Aldrich), supplemented with 5% FBS (GIBCO), 2 nM triiodothyronine (Sigma), 0.5% insulin-transferrin-selenium reagent (Life Technologies), 5 μg/ml transferrin (Life Technologies), 10 ng/mL epidermal growth factor (Sigma), 0.4 μg/mL hydrocortisone (Sigma), 1 nM cholera toxin (List Biological Labs), 0.5 μg/mL amphotericin B (Fungizone; Bristol-Myers Squibb), 40 μg/mL gentamicin (Gentacin; Life Technologies), 50 nM calpeptin (ENZO Life Sciences), 40 ng/ml Recombinant Human IL-1RA (PeProTech), 3 μg/ml Recombinant Human R-Spondin-1 (PeProTech). All cells were maintained at 37°C in a humidified incubator, with 5% CO2. The cell growth curves were plotted as accumulated population doublings versus time (days) [15]. The cell line generated in primary epithelial culture medium was referred to human normal bronchial epithelial cells (HNBEC).

### 2. Chemicals and regents

10~20nm-sized SiO_2_, and Cr(VI), B(a)P were purchased from Sigma corporation (USA). Actinomycin D was purchased from Roche (Switzerland). The kits for cell apoptosis and cell counting kit-8 (CCK-8) were purchased from Dojindo Molecular Technologies, Inc. (Kumamoto,Japan). Rabbit polyclonal antibody against p21 and mouse monoclonal antibodies against p53 and Actin were from Santa Cruz Biotechnology (Santa Cruz, CA).

### 3. STR analysis

HNBEC cellular genome DNA was isolated with the kit (Tiangen, Beijing). Short tandem repeat (STR) analysis (DNA fingerprinting) was performed using a commercial kit (PowerPlex 16 HS System; Promega Corporation, Madison, WI). This system allows the co-amplification and three-color detection of 16 loci (15 STR loci and the Y-chromosome–specific Amelogenin). The PCR amplification was performed according to the manufacturer’s recommended protocol with the ABI 3100 genetic analyzer (Applied Biosystems). Data analysis and allele size determination were performed using Genotyper and PowerTyper16 Macro Software (Applied Biosystems).

### 4. Karyotype analysis

Exponentially growing HNBEC cells were treated with 0.2 μg/ml colchicine for 3.5 hrs. Cells were then collected and continued with hypotonic treatment and fixation. Metaphase spreads were prepared and stained to observe chromosome. Twenty metaphase spreads were analyzed and photographed under the microscope.

### 5. Three-dimensional culture

Single-cell suspensions of HeLa and HNBEC cells were prepared into appropriated differential medium (keratinocyte growth medium, Invitrogen) containing 2% Matrigel (BD Biosciences). Morphogenesis assays were performed as previously described [16, 17].

### 6. Western blot analysis

HNBEC cells were treated with or without actinomycin D for 24 hrs and then harvested with modified radioimmuno-precipitation assay (RIPA) buffer as described in the early study [18]. Fifty microgram of total extract was subjected to electrophoresis and transferred onto polyvinylidene difluoride (PVDF) membranes (Millipore). The membranes were probed with specific antibodies described in Chemicals and reagents. Blots were developed using an enhanced chemiluminescence reagent (Beyotime).

### 7. Xenograft Assays

Exponentially growing HNBEC or HeLa cells (2x10^6^) were trypsinized, dispersed into single cells. The cells were resuspended with saline and injected s.c. into the back flanks of 6-week-old male mice with severe combined immunodeficiency (Guangdong medical laboratory animal center, Guangzhou). A total of three mice were injected for each cell types. Animal housing and sacrificing was in accordance with guidelines of the ARRIVE (Animal Research: Reporting of In Vivo Experiments). The Animal Ethics Committee of Wuhan University Shenzhen Institute and Shenzhen Center for Disease Control and Prevention approved this study. The growth of xenografts was observed daily. When the xenograft present in a mouse reached 1.6 cm^3^ in size, the mouse was euthanized using CO_2_ and xenograft tissue was isolated and remov-ed for histology.

### 8. Cell culture and the treatment with SiO_2_, Cr(VI), B(a)P

HeLa cells were cultured in DMEM supplemented with 10% FBS (Invitrogen) and 1% penicillin/streptomycin. Human bronchial epidermal cells (16HBE) immortalized by SV40 large T-antigen were described in the early studies [3, 19]. 16HBE cells were cultured in MEM medium supplemented with 10% FBS (Invitrogen) and 1% penicillin/streptomycin.

The SiO_2_ particles were processed by sonication. Cr(VI) was dissolved in sterile deionized H2O. B(a)P was dissolved in DMSO. The final concentrations of SiO_2_ particles were 40 μg/ml, 80 μg/ml, 120 μg/ml, 160 μg/ml, 200 μg/ml. The final concentrations of Cr (VI) were 5 μM, 10 μM, 15 μM, 20 μM, 30 μM. The final concentration of B(a)P were 20 μM, 40 μM, 80 μM, 100 μM, 120 μM. Cells were cultured to reach 80% confluence and treated with different concentrations of SiO_2_ particles、Cr(VI)、B(a)P for 24 hrs. The morphology of cells was observed and photographed under a phase-contrast microscope.

### 9. Assay of cell viability

HNBEC cells and 16HBE cells were plated into a 96-well plate, and the cells were treated with nano-SiO_2_, Cr (VI) and B(a)P at the indicated concentration for 24 hrs when the cell confluence reached up to 80%. After the treatment, the cells viability was determined by CCK-8 kit according to the manufacturer’s recommended protocol. The absorbance was measured at 450 nm using a microplate reader (BioTek, Winooski, VT, USA). The reference wavelength is 630 nm. The inhibitory rate (IR) of the cell growth was figured out by the formula provided by the kit. The IC50 value was therefore determined.

### 10. Cell apoptosis detection

The apoptosis of HNBEC cells and 16HBE cells were measured as described in the instruction provided by the Annexin V-FITC apoptosis detection kit. In brief, after a 24 h treatment, about 1~5 x 10^5^ cells were collected and washed with PBS (centrifuged at 2,000 rpm for 5 min). The cells were resuspended in 500 μl binding buffer, and then added by 5 ul of Annexin V-FITC and 5 ul of PI, and incubated in darkness at room temperature for 5~15 min. The cells were analyzed in a flow cytometer (Becton Dickinson, USA).

### 11. Statistical analysis

Data were shown as mean ± SD and analyzed using SPSS 13.0 statistical software (SPSS Inc., Chicago, Illinois, USA). The One-Way ANOVA procedure followed by Student-Newman-Keuls test was used to determine the different means among groups. The level of significance was set at p < 0.05.

## Results

### 1. Generation of human normal bronchial epithelial cells (HNBEC)

**Fig 1:**
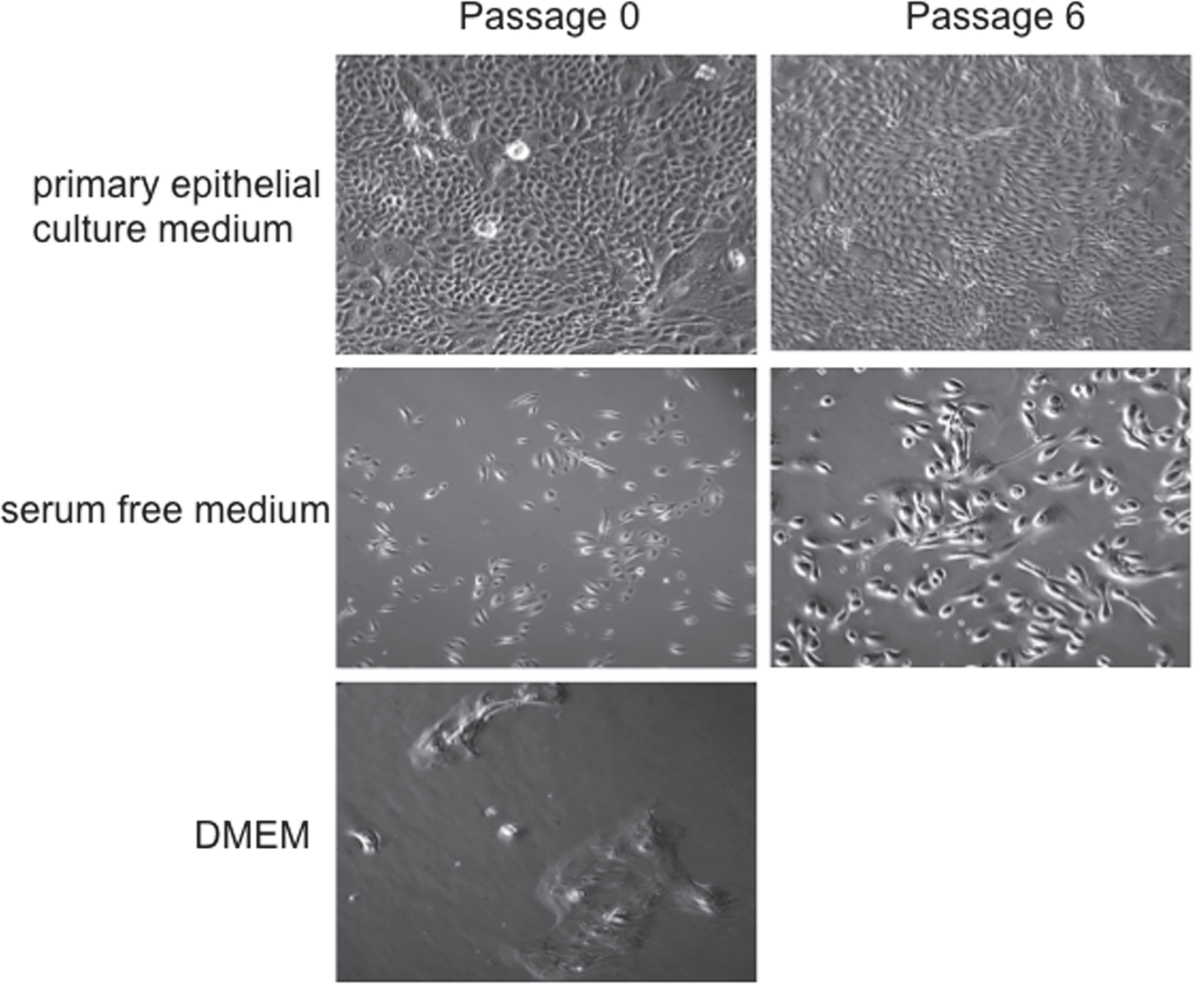
Morphology of human normal bronchial epithelial cells (HNBEC) in different culture conditions. Lung tissues were harvested and digested with trypsin-collagenase, and then grown in primary epithelial culture medium, or serum free medium, or DMEM as described in Materials and Methods. Small cell clusters formed after 1 day in primary epithelial culture medium. The photographs were taken at passage 0 and 6 at a 100×magnification. Cells did not proliferate in DMEM.

**Fig 2:**
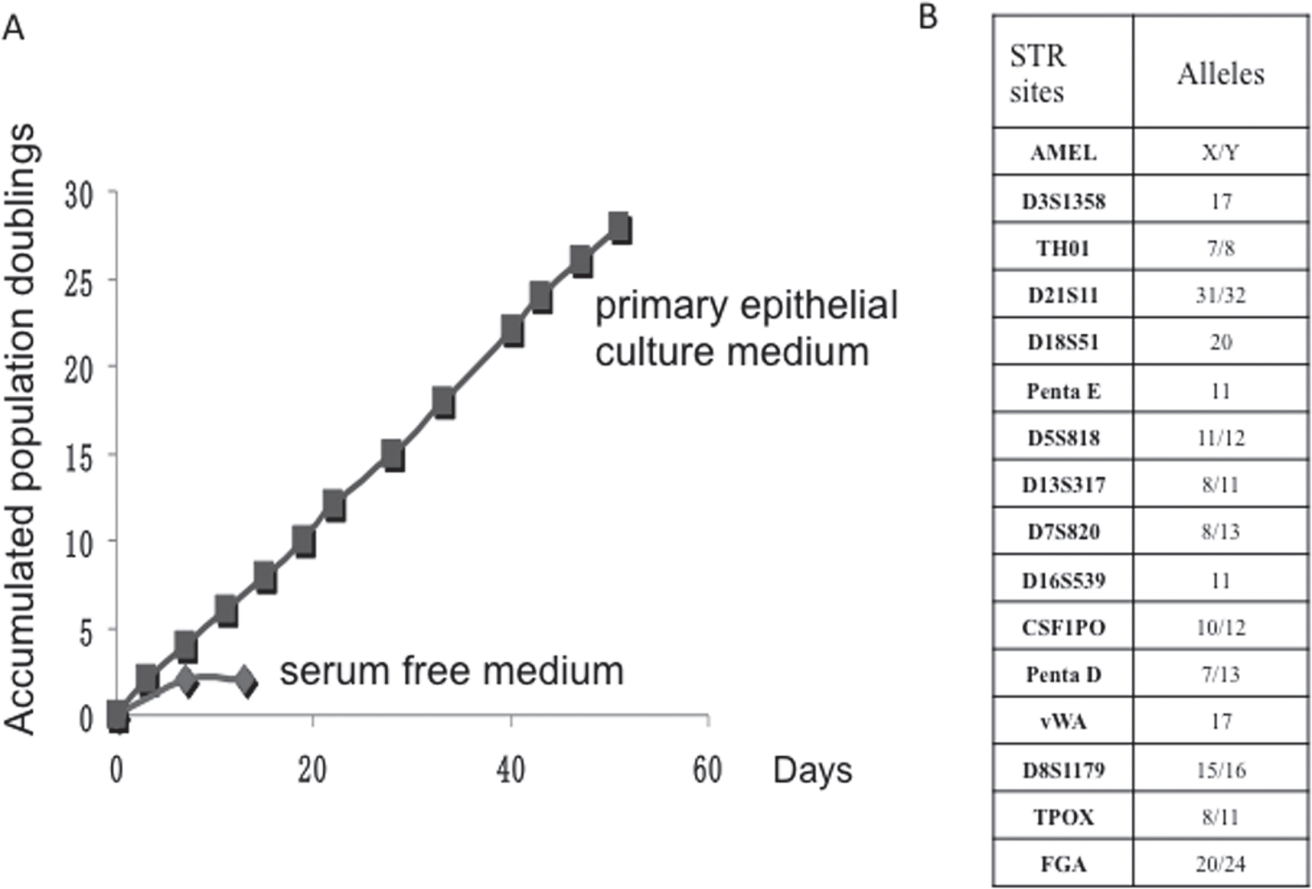
Propagation of human normal bronchial epithelial cells (HNBEC). (A) HNBEC cells were passaged repetitively using trypsinization technique in primary epithelial culture medium or serum free medium. The cell number was recorded at each passage, and a plot of accumulated population doublings versus time (days) was constructed. (B) DNA fingerprinting of HNBEC cells were analyzed by STR analysis. HNBEC cells have 15 STR loci and the Y-specific Amelogenin locus that do not match any other existing cell lines.

The histology of the mirrored specimen confirmed that there was no contamination of tumor cells within the specimen for the primary culture. Isolated cells were cultured in primary epithelial culture medium, or serum free medium, or DMEM as described in Materials and Methods. Epithelial small colonies were observed after 1 day of plating and expanded rapidly within 8 days in primary epithelial culture medium. In contrast, cells proliferated slowly in serum free medium and did not grow in DMEM (Fig 1). After the first plating, HNBEC cells were passaged every 3–4 days using trypsinization technique as described in Materials and Methods. The cell number was recorded at each passage, and a plot of accumulated population doublings versus time (days) was constructed. Fig 2A showed constant growth rate of HNBEC cells in primary epithelial culture medium during the cultured 51 days with 28 accumulated population doublings. In contrast, cells propagated for the very limited passages and only reached 2 population doublings in serum-free medium. To verify the uniqueness of HNBEC cells, we performed short tandem repeats (STR) analysis (DNA fingerprinting) (Fig 2B). The result showed that HNBEC cells have 15 STR loci and the Y-specific Amelogenin locus. STR analysis confirmed that HNBEC cells were generated from a specific individual and did not match any other cell lines published or registered before.

### 2. Characterization of normal biology of HNBEC cells

**Fig 3:**
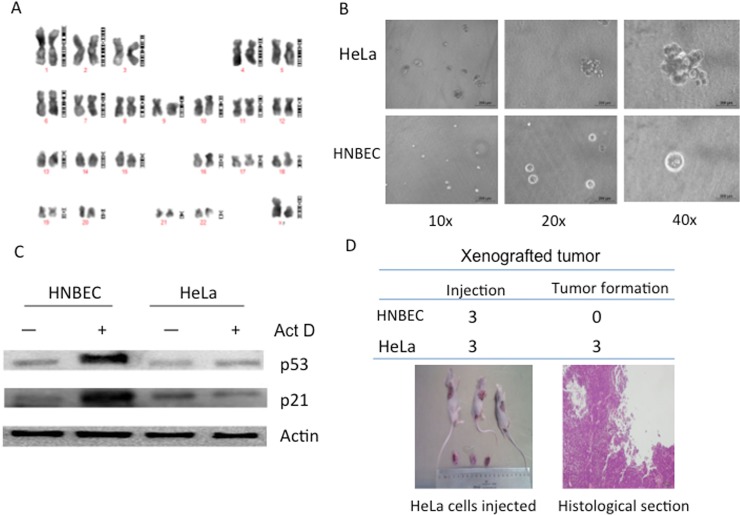
Characterization of HNBEC cells. (A) Normal Karyotype of HNBEC. Twenty metaphase spreads were analyzed and photographed under the microscope. An image of a normal karyotype (46, XY) of cells was shown. (B) Differentiation potential of HNBEC cells. Single-cell suspensions of HeLa and HNBEC cells were cultured in medium containing 2% Matrigel. Morphogenesis assays were performed and photographed. (C) DNA damage response by treatment of cells with 0.5nM actinomycin D (Act D). HNBEC, HeLa cells were treated by actinomycin D for 24 hrs. The responses of the cells to Act D were measured by immunoblot analysis of p53 levels and its downstream target p21. Actin serves as a loading control. The experiment was repeated three times, the representative data were shown. (D) Xenograft assays. A total of three mice were injected with either HNBEC or HeLa cells. HNBEC cells did not induce any tumors in mice, while HeLa cells induced tumors in all three mice. The induced tumors and a representative H&E staining histological section were shown.

First, we wanted to see whether these cells harbored any abnormal karyotypes in culture, a conventional cytogenetic analysis demonstrated that HNBEC cells exhibited a structurally and numerically normal karyotype with a 46, XY (Fig 3A). Secondly, we would like to evaluate the differential potential of these cells, we studied this potential using Matrigel three-dimensional (3D) culture. As shown in Fig 3B, HNBEC cells formed well-defined spheres when cultured in differention medium. In contrast, HeLa cells (a cervical cancer cell line) formed solid masses of disorganized aggregates. In most cancer cell lines and exogenous gene-immortalized cells, the signaling pathways (such as the aberrant p53 and/or Rb regulatory pathways) are always altered to enable cells to survive in vitro. To study whether HNBEC cells have a normal p53-induced growth pathway and are able to respond to DNA damage normally, we treated the cells with actinomycin D (Act D) for 24 hrs. HeLa cells were used as a control. Act D induces DNA strand breaks and a p53-mediated growth arrest in normal cells [15]. As shown in Fig 3C, p53 protein level increased in HNBEC cells treated with Act D and the downstream effector p21^CIP1^ was also upregulated compared to untreated HNBEC cells. However, p53 protein level was not induced and neither p21^CIP1^ was upregulated in HeLa cells with Act D treatment. Therefore, these data indicated that HNBEC cells possessed a normal cellular function to mediate DNA damage responses. To further verify the normal biological character of HNBEC cells in vivo, we carried out the xenograft assays. HeLa cells and HNBEC cells were suspended with saline and injected subcutaneously into the back flanks of 6-week-old male mice with severe combined immunodeficiency. After two weeks, HeLa cells were able to induce tumors in three mice out of three while HNBEC cells were not able to induce any tumor in three mice (Fig 3D). The tumor-bearing mice were sacrificed and the H&E staining histological section indicated the tumor tissue induced by HeLa cells.

### 3. Morphological changes of HNBEC and 16HBE cells exposed to SiO_2,_ Cr(VI) and B(a)P

**Fig 4:**
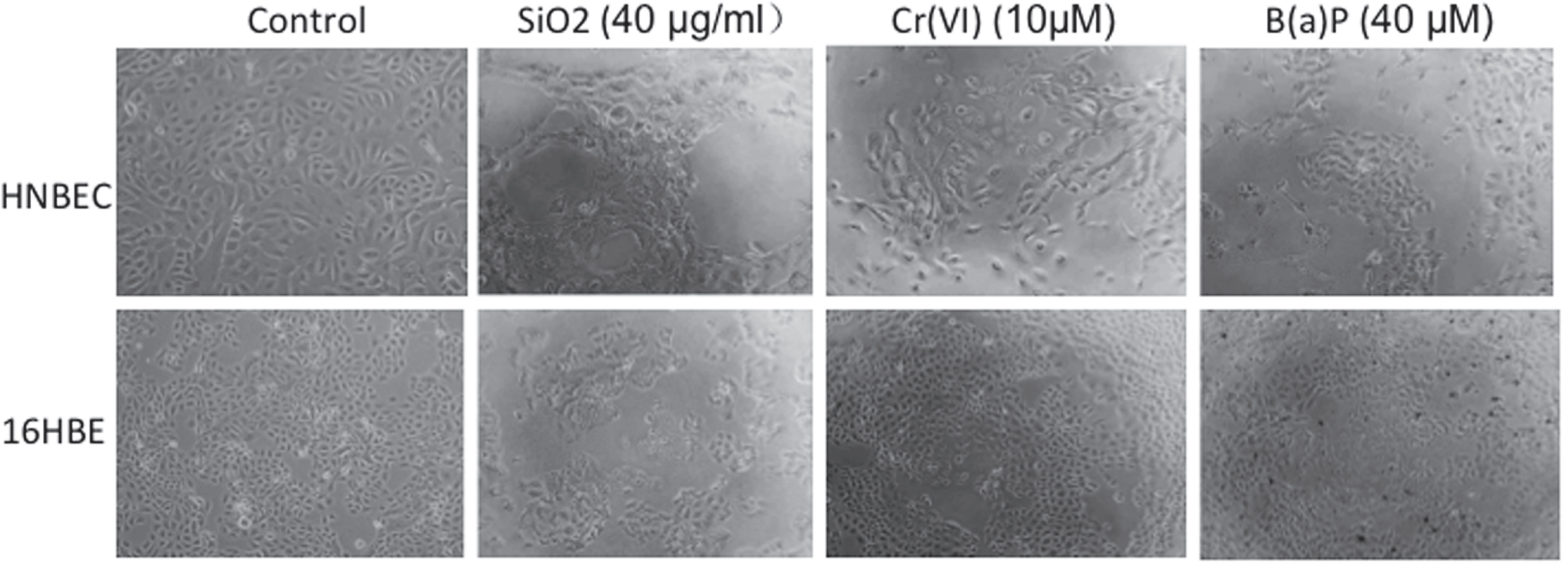
Morphological changes induced by SiO_2,_ Cr(VI) and B(a)P exposure. HNBEC and 16HBE cells were exposed to 10~20nm-sized SiO2, Cr(VI) and B(a)P for 24 hrs with concentrations of 40 μg/ml, 10μM and 40 μM, respectively. The morphology of cells was observed and photographed at a 100× magnification.

Human bronchial epidermal cells (16HBE) were described as a cell line immortalized by SV40 large T [3, 19]. 16HBE cells have been used to evaluate the cytotoxicity of Chromium (VI) and Benzo(a)pyrene in the earlier studies [3, 4]. We used 16HBE cells as the control cells and evaluated the toxicity of SiO_2,_ Cr(VI) and B(a)P exposure on HNBEC cells. We treated HNBEC and 16HBE cells with 10~20 nm-sized SiO_2_, Cr(VI) or B(a)P at concentrations of 40 μg/ml, 10 μM or 40 μM, respectively. After 24 h-exposure, more than 50% of HNBEC cells detached in response to three toxicants (Fig 4). In contrast, less than 30% of 16HBE cells detached when treated with 10~20nm-sized SiO_2._ Exposure to Cr(VI) and B(a)P mainly resulted in irregular cellular shapes in 16HBE cells. These data indicated that the morphological damages were more severe to HNBEC cells than to 16HBE cells for all three toxicants tested (Fig 4).

### 4. Cell viability changes of HNBEC and 16HBE cells exposed to SiO_2,_ Cr(VI) and B(a)P

**Fig 5:**
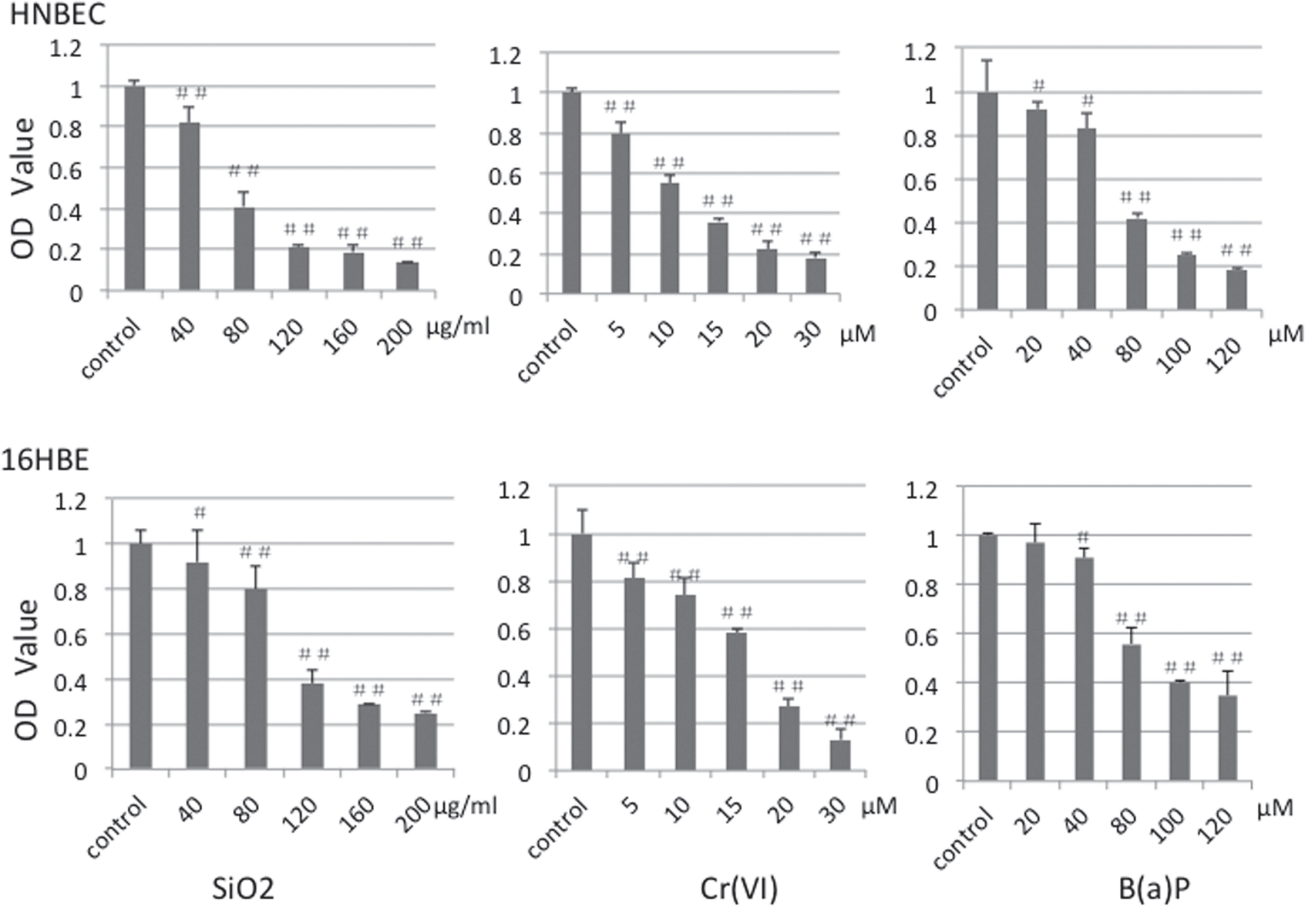
Viability of HNBEC and 16HBE cells after 24 hrs exposure to SiO_2,_ Cr(VI) and B(a)P. HNBEC and 16HBE cells were treated with 10~20nm-sized SiO_2_, Cr(VI) and B(a)P for 24 hrs at indicated concentrations. After the treatment, the cells were incubated with CCK-8 for 2 hrs, the plate was read at 450 nm. Values were mean ± SD from three independent experiment. *#* p*<0*.*05*, *##* p*<0*.*01* vs control cells.

Besides the morphological alterations, exposure to 10~20 nm-sized SiO_2_, Cr (VI) and B(a)P resulted in significantly decreased cell viability in both HNBEC and 16HBE cells (Fig 5). HNBEC cells were treated with 10~20 nm-sized SiO_2_ at concentrations of 40μg/ml, 80 μg/ml, 120 μg/ml, 160 μg/ml, 200 μg/ml. After 24 hrs, cell viability of HNBEC cells dramatically decreased in a dose-dependent manner. The relationship of inhibitory rate of the cell growth and the dosages of SiO_2_ was analyzed by SPSS 13.0 software and the IC50 (50% concentration of inhibition) value of SiO_2_ was 44 μg/ml. 16HBE cells were treated with 10~20 nm-sized SiO_2_ at the same gradual concentrations as those of HNBEC cells. The IC50 value of 16HBE cells exposed to SiO_2_ was 84.3 μg/ml. Treatment of Cr(VI) was carried out at concentrations of 5 μM, 10 μM, 15 μM, 20 μM, 30 μM and B(a)P was at concentrations of 20 μM, 40 μM, 80 μM, 100 μM, 120 μM. The IC50 values of HNBEC and 16HBE cells exposed to Cr(VI) were 11.6 μM and 15.9 μM, respectively. The IC50 values of HNBEC and 16HBE cells exposed to B(a)P were 60.7 μM and 91.3 μM, respectively. These results indicated that HNBEC cells were more sensitive to exposure of three toxicants than 16HBE cells. One interesting observation was that exposure to inhaling toxicants, 10~20 nm-sized SiO_2_ and B(a)P, resulted in much more significant difference in cell viability between HNBEC and 16HBE according to the IC50 values. Therefore, we chose 10~20 nm-sized SiO_2_ as the testing toxicant exposed to the bronchial epithelial cells (both HNBEC and 16HBE) in the subsequent experiments.

### 5. Apoptosis of HNBEC and 16HBE cells exposed to SiO_2,_ Cr(VI) and B(a)P

**Fig 6:**
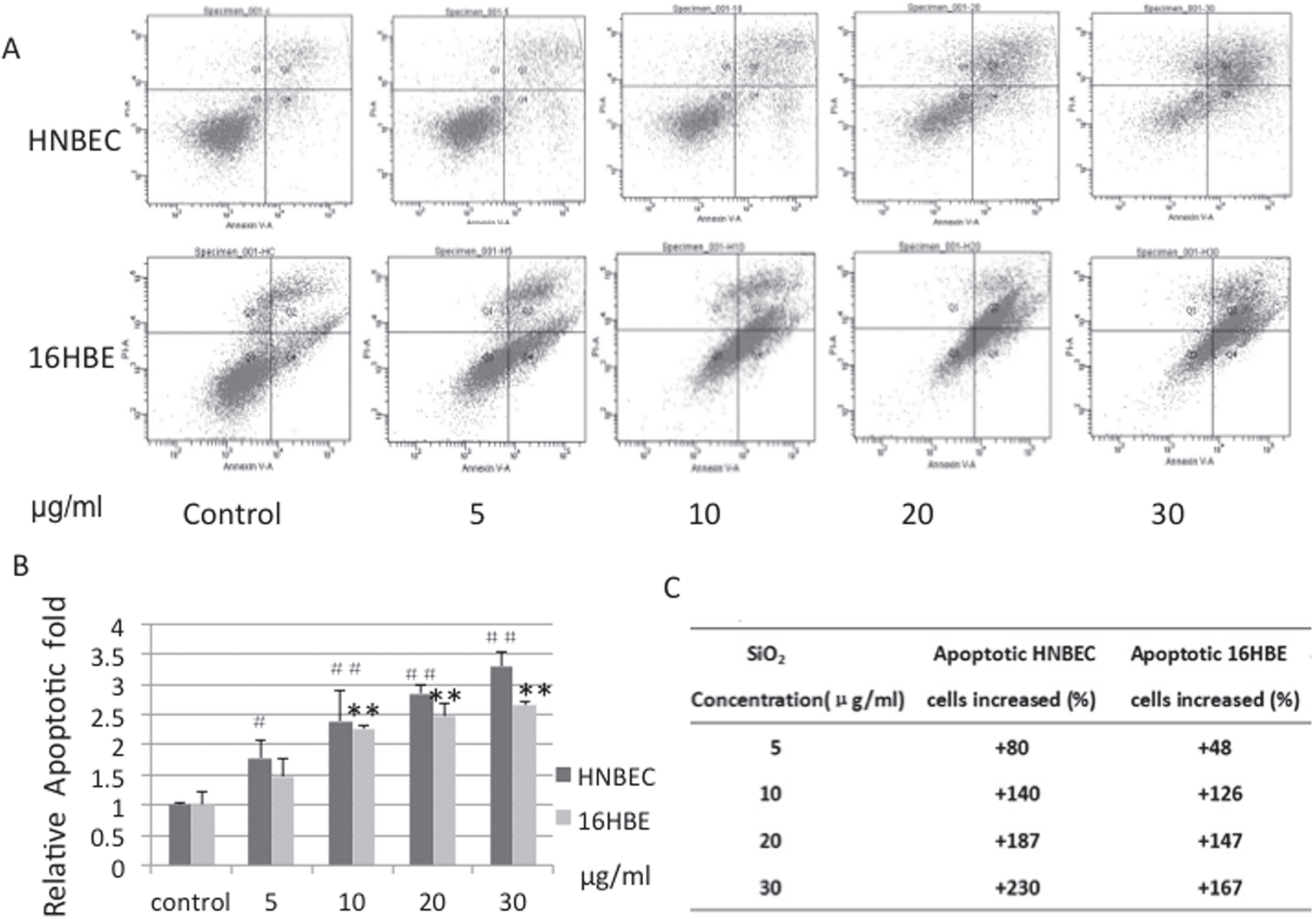
The effect of exposure to SiO_2_ on cellular apoptosis of HNBEC and 16HBE cells. (A) The representative images of apoptosis of HNBEC and 16HBE cells after 24 hrs exposure to 10~20 nm-sized SiO_2_ at the dosages of 5 μg/ml、10 μg/ml、20 μg/ml、30 μg/ml. Quadrant Q1、 Q2 、Q3 and Q4 represent the portion of necrotic cells, late-stage apoptotic cells, normal cells, and early-stage apoptotic cells, respectively. (B) The quantitative results of above apoptotic images. The ratio of apoptotic cells versus normal cells in HNBEC and 16HBE were normalized by each cell types itself. Values were mean ± SD from three independent experiments. ## and ** indicate p <0.01 vs control cells; # indicates p < 0.05 vs control cells exposed to SiO_2_ particles. (C) The quantitative results of apoptotic cells increased in HNBEC and 16HBE by percentile score were shown.

Since the IC50 value of HNBEC cells exposed to 10~20 nm-sized SiO_2_ was 44 μg/ml, the dosage of 30 μg/ml (about 2/3 IC50) was used as the maximum dosage in apoptosis analysis. Flow cytometry analysis indicated that exposure to 10~20 nm-sized SiO_2_ induced apoptosis of both HNBEC and 16HBE cells in a dose-dependent manner (Fig 6A). Interestingly, the apoptotic pattern was different between HNBEC cells and 16HBE cells. Exposure to low concentration of SiO_2_ (5 μg/ml), late-stage apoptotic HNBEC cells were increased rapidly compared to early-stage apoptotic cells (Fig 6A). As the concentration of SiO_2_ increased, late-stage apoptotic HNBEC cells were still more readily observed than early-stage apoptotic cells. In the case of 16HBE cells, exposure to low concentration of SiO_2_, more early-stage apoptotic cells were observed than late-stage apoptotic cells (Fig 6A). As the concentration of SiO_2_ increased, the transition from early-stage apoptotic 16HBE cells to late late-stage apoptotic cells was observed. The apoptotic rate increased gradually while the concentrations of SiO_2_ increased (p<0.05 or p<0.01, Fig 6B). The quantitative results of apoptotic cells increased were shown in Fig 6C. The results indicated that HNBEC cells were more sensitive than 16HBE cells to exposure of 10~20 nm-sized SiO_2._

## Discussion

Due to the potential primary route of exposure to environmental toxicants, human epithelial cells become major concern of damaging targets. The organ-specific differentiated epithelial cells carry out the vital functions, for example, gas exchange in the lung, filtration in the kidney, detoxification and conjugation in the liver, insulin production in the pancreatic islet cells or protection against a hazardous environment by the skin. Therefore, epithelial-based in vitro models have been used to investigate the mechanisms of occupational exposure to toxicants [1–4, 10, 12, 20]. Among these studies, cancer cell line A549, HT-29 or aneuploid chromosomal cells are used and arise some controversial results against each other [1, 2, 12, 21]. To avoid the usage of cancer cells, viral or cellular oncogenes have been transduced into cells to establish the “normal” cells in vitro [21–23]. However, genetic manipulations change the cells’ genetic background as well as physiology such that cells may not resemble or function like normal epithelial cells [13, 15].

In this study, we established human normal bronchial epithelial cells (HNBEC) from the patient derived lung tissue without transduction of any exogenous genes. We compared the different conditions for the primary culture. Fig 1 showed the propagation and morphologies of HNBEC cells in primary epithelial culture medium, serum free medium and DMEM. HNBEC cells grew slowly in serum free medium and did not grow in DMEM. That is the reason why even gene-immortalized epithelial cells are cultured in serum free medium supplemented with individual growth factors. However, the culture of primary epithelial cells has been proved to be difficult over the years. HNBEC cells could not bypass the senescence and expand slowly in serum free medium. While HNBEC cells proliferated rapidly in primary epithelial culture medium (Fig 1) and maintained a constant growth rate until observed 28 accumulated population doublings (Fig 2A). Recently, two groups established a method to indefinitely extend the life span of primary human keratinocytes and epithelial cells using murine fibroblastic 3T3 feeder cells and a Rock inhibitor [13, 15]. Despite of these inspiring findings in cell culture technology, the exact mechanism for promoting unrestricted cell proliferation is still unclear. It has been speculated that the induction of telomerase, cytoskeletal remodeling and/or interference with the p16/Rb pathways are the critical required functions [13, 15]. Based on screens for cytokines with epithelial cell proliferation activity, Kondo et al reported that IL-1α inhibits the growth of epithelial cells, whereas IL-1 receptor antagonist (IL-1RA) promotes growth [24]. Recently, Hans Clevers group[25, 26]established a three-dimensional culture system which allows long-term clonal expansion of single Lgr5(+) stem cells into transplantable organoids (budding cysts), these cells retain many characteristics of the original epithelial architecture. A crucial component of the culture medium is the Wnt agonist RSPO1, the recently discovered ligand of LGR5 which is Wnt target gene. We speculate that primary epithelial cell culture medium in this study extends life span of human normal bronchial epithelial cells through cooperation of decreased IL-1 signaling and increased Wnt signaling.

We also characterized the genetic and biological aspects of HNBEC cells. The DNA fingerprinting result (Fig 2B) showed that HNBEC cells are newly established cell line not matching any other cell lines published or registered before. The typical diploid karyotype of HNBEC cells indicated that cellular chromosomes are structurally and numerically normal, with a 46, XY karyotype (Fig 3A). Three-dimensional culture indicated that HNBEC cells formed normal spheres in differential medium compared to those solid masses in HeLa cells (Fig 3B). This matrigel 3D culture system has been widely used to distinguish normal from malignant epithelial cells[17] [27]. Moreover, HNBEC cells possessed a normal cellular function to mediate DNA damage responses (Fig 3C). The xenograft assays indicated that HNBEC did not induce any tumor in vivo while HeLa cells did (Fig 3D). Taken together, HNBEC cells retain the normal biological and genetic features. Thus, HNBEC cells have advantages for many research areas compared to the spontaneously immortalized keratinocytes (HaCaT cells with an aneuploid chromosome) [11] and cancer cell lines or viral/oncogene immortalized cell lines since those cells have an altered genetic background/signaling pathways and exhibit an abnormal biological characters [13] [14]. We believe that the culture system in this study not only facilitate expansion of human bronchial epithelial cells, but may also benefit for expanding life span of other types of mammalian epithelial cells and will have significant impacts in cell biology, tumor biology, drug discovery and safety evaluation, and regenerative medicine as well.

In this study, we evaluated the potential applications of HNBEC cells in study of the common occupational toxicants exposure to lung tissue as a novel in vitro human cells-based toxicological model. Human bronchial epidermal cells (16HBE), a viral gene-immortalized cell line, were used as control cells. Exposure to SiO_2,_ Cr(VI) and B(a)P causes more severe morphological changes in HNBEC cells than those in 16HBE cells (Fig 4). Cell viability analysis showed that HNBEC cells were more sensitive to exposure of three toxicants than 16HBE cells (Fig 5). Moreover, the inhaling toxicants, 10~20 nm-sized SiO_2_ and B(a)P, resulted in more significant difference in cell viability between HNBEC and 16HBE according to the IC50 values. Flow cytometry analysis also showed that HNBEC cells were more sensitive than 16HBE cells to exposure of 10~20 nm-sized SiO_2_ but the apoptotic patterns were different between these two cell lines (Fig 6A). The late-stage apoptotic HNBEC cells were more readily observed than early-stage apoptotic cells at the exposure to low concentration of SiO_2_. Since HNBEC cells were established without any genetic background changes, the cellular responses of these cells to toxicants should be closer to physiological status. Our results demonstrated that HNBEC cells are a novel and valuable in vitro cell model for toxicity evaluation. Importantly, we also need to consider the tissue origin of epithelial cells according to the potential primary route of exposure, such as inhaling toxicants versus lung tissue. Different organ-specific normal epithelial cells need to be established to evaluate the real physiological responses to toxicants in the near future. In terms of 3D cultures that mimic in vivo environment, that would be interesting to explore how these functional normal cells behave with 3D cultures and how these cells respond to the common environmental toxicants. The combination of 2D and 3D human cells based cultures may be a great replacement or addition to the current cell lines and/or animal based system for toxicity evaluation and drug discovery.
